# A Low-Cost Approach to Automatically Obtain Accurate 3D Models of Woody Crops

**DOI:** 10.3390/s18010030

**Published:** 2017-12-24

**Authors:** José M. Bengochea-Guevara, Dionisio Andújar, Francisco L. Sanchez-Sardana, Karla Cantuña, Angela Ribeiro

**Affiliations:** 1 Centre for Automation and Robotics, CSIC-UPM, Arganda del Rey, Madrid 28500, Spain; jose.bengochea@csic.es (J.M.B.-G.); dionisioandujar@hotmail.com (D.A.); fl.sanchez@csic.es (F.L.S.-S.); karlacantunaflores@yahoo.es (K.C.); 2 Institute of Agricultural Sciences, CSIC, Madrid 28006, Spain; 3 Departamento de Ingeniería Informática y Sistemas Computacionales, Cotopaxi Technical University, Latacunga 050101, Ecuador

**Keywords:** 3D reconstruction, RGB-D sensor, crop inspection platform

## Abstract

Crop monitoring is an essential practice within the field of precision agriculture since it is based on observing, measuring and properly responding to inter- and intra-field variability. In particular, “on ground crop inspection” potentially allows early detection of certain crop problems or precision treatment to be carried out simultaneously with pest detection. “On ground monitoring” is also of great interest for woody crops. This paper explores the development of a low-cost crop monitoring system that can automatically create accurate 3D models (clouds of coloured points) of woody crop rows. The system consists of a mobile platform that allows the easy acquisition of information in the field at an average speed of 3 km/h. The platform, among others, integrates an RGB-D sensor that provides RGB information as well as an array with the distances to the objects closest to the sensor. The RGB-D information plus the geographical positions of relevant points, such as the starting and the ending points of the row, allow the generation of a 3D reconstruction of a woody crop row in which all the points of the cloud have a geographical location as well as the RGB colour values. The proposed approach for the automatic 3D reconstruction is not limited by the size of the sampled space and includes a method for the removal of the drift that appears in the reconstruction of large crop rows.

## 1. Introduction

Precise crop monitoring helps farmers improve crop quality and reduce operational costs by making better crop management decisions. Thus, yield estimation is typically based on crop knowledge, historical data, meteorological conditions and crop monitoring that is generally conducted via manual sampling. However, manual sampling is a time-consuming, labour-intensive, and frequently inaccurate process, mainly because the number of samples is often too small to capture the magnitude of variations in a crop, particularly for regions of more than several hectares. Thus, it is extremely important to identify an automated and efficient alternative to manual sampling that can accurately capture the spatial and temporal variations of a crop. Towards this end, vehicles equipped with on-board sensing equipment are a promising choice among the various means of collecting well-structured information. Furthermore, the use of medium-sized platforms for full crop scouting is a suitable choice for minimising soil compaction that, among other applications, enables the performance of more than one sampling throughout the year due to having minimal impacts on the crop.

Alternatively, 3D reconstruction of woody crop models using non-destructive methods is a valuable technique to improve the decision-making processes. The use of sensors for the characterisation of crops leads to a better understanding of the processes involved in tree development throughout their life cycle. With information obtained from a 3D reconstruction of the crop, important parameters, such as the growth status, height, shape, biomass, need for nutrients, and health status, can be estimated. These parameters are currently mostly estimated by applying equations that assume the trees to be geometric solids (regular polygons) or by applying empiric models [1], which produce inconsistent results. The use of the information extracted from 3D reconstructions can improve the decisions made related to crop management and contribute to creating new protocols to improve the profitability and health of plants.

RGB-D sensors are one of the most promising solutions on the market to obtain data for reconstructing 3D models of a scene. In particular, the Microsoft Kinect v2 sensor has assumed a leading role in recent years because of its low cost and favourable performance. This sensor, based on time-of-flight (ToF) technology, provides additional information to the RGB data, i.e., depth, infrared and skeleton frames that, among other applications, have been used to characterise plants in agriculture. Thus, in [2], the authors compared two low-cost 3D systems, including the Kinect v2 sensor, with an expensive high-precision laser scanner and concluded that the low-cost systems can replace the more expensive scanner in several plant phenotyping scenarios. The use of the Kinect v2 sensor was proposed in [3] to determine the volume of weeds in maize crops and to define their treatment period. The results suggest that this sensor can serve as a high-precision device in estimating the volume of weeds and determining the state of the crop. In [4], a Kinect v2 sensor was used to estimate the volume of onions, showing that the calculated volume was directly related to the estimation based on the measurements from the sensor. As a result, although 3D reconstruction is a research topic associated with numerous and important results involving computer vision, the emergence of sensors, such as the above-mentioned sensors, which provide information on the distance to the objects closest to the scene (depth data), has enabled new possibilities in 3D reconstruction. Several recent research studies have focused on acquiring 3D scene reconstruction using the depth images supplied by these new sensors. Furthermore, various techniques have been proposed that employ distinct types of accelerated data structures using graphics hardware to combine consecutive depth images with a certain degree of overlap. Each technique has its own advantages and disadvantages in terms of speed, scale and quality of reconstruction.

Certain methods use the voxel structure, which represents a value on a regular grid in 3D space to store 3D sensor information [5,6,7]. A well-known example is the method described in [6], which generates high-quality 3D reconstructions [8] and was adopted by Kinect Fusion [9,10], i.e., it is the 3D reconstruction method included by Microsoft in its software development kit—Kinect for Windows Software Development Kit 2.0 [11]. However, the method has the important constraint of not allowing the reconstruction of large scenarios with volumes larger than 8 cubic metres. This constraint has motivated the emergence of variants on the method that allow reconstructions of larger volumes using voxel structures [12,13,14], while other strategies are based on the use of hierarchical data structures, which divide the space effectively but are not easily parallelized, thus resulting in added computational complexity [15,16].

One of the limitations of 3D reconstruction methods is that they estimate the position and orientation of the sensor with the information obtained from the set of images, i.e., slight variations can exist between the calculated position and orientation and the actual values obtained by the sensor. Such differences arise primarily with similar scenes, such as crops, in which the same structure is repeated, appearing as analogous information (e.g., canopies full of similar leaves existing at similar distances from the sensor). These slight variations in calculated position and orientation can give rise to drift that causes deformations in the 3D reconstruction, with the deformations being more pronounced with the greater size of the reconstruction.

The overall objective of the present work is to provide a method to automatically generate 3D reconstructions of large zones, such as a complete crop row, from the information directly supplied by the Microsoft Kinect v2 sensor on-board a vehicle. The presented method includes a technique to manage the drift that appears in the reconstruction, which is accentuated with the line length.

## 2. Materials and Methods

### 2.1. The Acquisition Equipment at the Field

The RGB-D sensor Kinect v2 (Microsoft, Redmond, WA, USA), which operates at 30 fps, supplies RGB images with a resolution of 1920 × 1080 pixels together with depth information with a resolution of 512 × 424 pixels. The depth data is obtained via a ToF system inside the sensor, which modulates a light source using a square wave. The sensor uses phase detection to measure the time for light to travel from the light source to the object and back to the sensor and then estimates the distance from the results. The system calculates the distance from the speed of light in air by estimating the received light phase at each pixel with knowledge of the modulation frequency. The depth measurement range of the sensor is 0.5–4.5 m [17]; outdoors, however, the maximum range decreases. Specifically, studies conducted outdoors under different daytime illumination conditions [18] show that the sensor provides valid depth measurements up to 1.9 m during sunny days, while the distance increases up to 2.8 m under the diffuse illumination of an overcast day.

**Figure 1:**
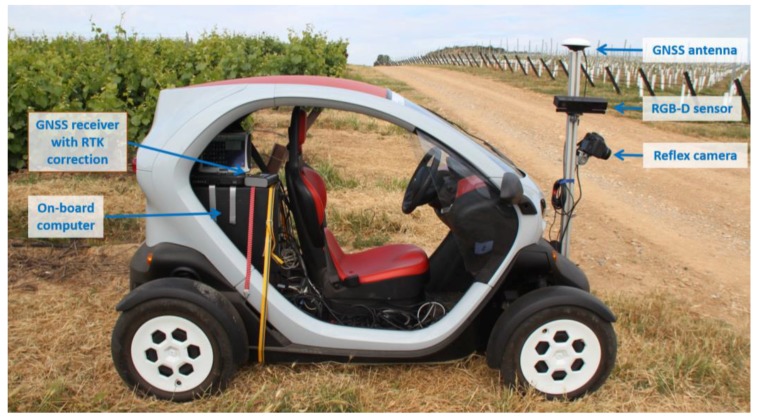
Field platform and on-board equipment.

The Kinect v2 sensor is placed in a field platform (Figure 1) using an aluminium support structure. The field platform is based on a Twizy Urban 80 model (Renault, Valladolid, Spain), which has a 13 kW electric motor and can travel up to 80 km/h. The vehicle is ultra-compact, with a length of 2.32 m, width of 1.19 m, height of 1.46 m and unladen weight of 450 kg; moreover, the completed battery charge allows a total travel distance of over 80 km. The electric motor of the vehicle allows negligible vibration at speeds below 3 km/h [19], which is convenient for high-quality information acquisition.

In addition to the Kinect v2, the platform is equipped with another sensor, a digital single-lens reflex camera (EOS 7D, Canon, Tokyo, Japan), which supplies high-quality RGB images at 2 fps with a resolution of 2592 × 1728 pixels. Both sensors are connected to the on-board computer, which contains an Intel Core i7-4771@3.5GHz processor, 16 GB of RAM, and an NVIDIA GeForce GTX 660 graphic card. The platform is also equipped with a RTK-GNSS receiver, an R220 receiver (Hemisphere, Scottsdale, AZ, USA), which provides location data at a 20 Hz sample rate with an accuracy of 20 mm + 2 ppm (2DRMS, 95%) according to the manufacturer’s specifications. The total cost of the described system was approximately 8000 euros, not including the reflex camera.

To illustrate the implications of a journey of 80 km (as determined by the autonomy of vehicle) in crop inspection, we can estimate the hectares that would be covered in the case of a woody crop inspected using RGB-D sensors. If the woody crop is a vineyard, then the space between crop rows (lane) is typically 2 m. If two RGB-D sensors are placed on both sides of the vehicle to record images of each row while the vehicle advances along the lane, the vehicle can cover a total of 16 ha. The covered area can be somewhat decreased, considering the consumption of energy of the equipment connected to the battery of the vehicle and the movements made in the crop headers to change lanes.

The inspection plan to be followed by the platform is generated by a path planner [20], which can be formulized as the well-known capacitated vehicle routing problem, as stated in [21]. The fundamental problem consists of determining the best inspection route that provides complete coverage of the field considering features (such as the field shape, crop row direction, and type of crop) and certain characteristics of the platform (such as the turning radius or the number of on-board sensors). Therefore, the planner determines the order for performing the lane analysis in such a manner that an established optimisation criterion is minimised.

While this mobile platform is prepared to inspect both annual (e.g., maize or cereal) and multi-annual (e.g., orchards or vineyards) crops, the present work is focused on the inspection and 3D reconstruction of woody crops. Note that in the case of arable crops, the platform can only scout the crop during the early season, which is acceptable since this coincides with the time when treatments for weeds are carried out.

### 2.2. 3D Reconstruction Approach

After studying different 3D reconstruction methods [12,13,14,16], the algorithm described in [14] was selected for the 3D reconstruction of woody crops, typically formed by several rows of a large length. This method provides satisfactory results in large-zone reconstruction from the information directly supplied by the Kinect sensor.

The method extends the algorithm proposed by [6] to reconstruct large regions using the fusion of different overlapped depth images, storing information only on the voxels closest to the detected object and accessing to the stored information using a hash table. In this way, a complete regular voxel grid stored in the memory is unnecessary, thereby providing computational advantages. Given a new input depth image and known camera position, the ray-casting technique [22] is used to project a ray from the camera focus for each pixel of the input depth image to determine the voxels in the 3D world that cross each ray. In this way, the voxels related to the depth information are determined.

**Figure 2:**
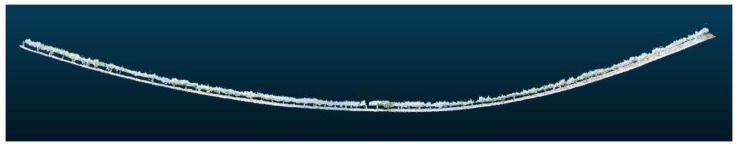
3D reconstruction of a woody crop row in which the drift problem is observed.

Once the surface of the scene has been extracted using the ray-casting technique, this information is used to estimate the position and orientation of the camera with 6 degrees of freedom when a new input image arrives. The last estimation is conducted with a variant of the iterative closest point (ICP) algorithm [23] and provides a point cloud as the output, which in this case is a woody row that does not appear to be as straight as the original row, showing detectable drift, as shown in Figure 2.

Finally, a method was designed and developed to accommodate this drift using the minimal scene information that can be acquired from the field platform during its travel, such as the geographical location of the starting and ending points of each sampling row and the fact that trees are typically planted in straight lines. In the following subsections, the developed method will be explained in detail.

#### 2.2.1. Filtering

**Figure 3:**
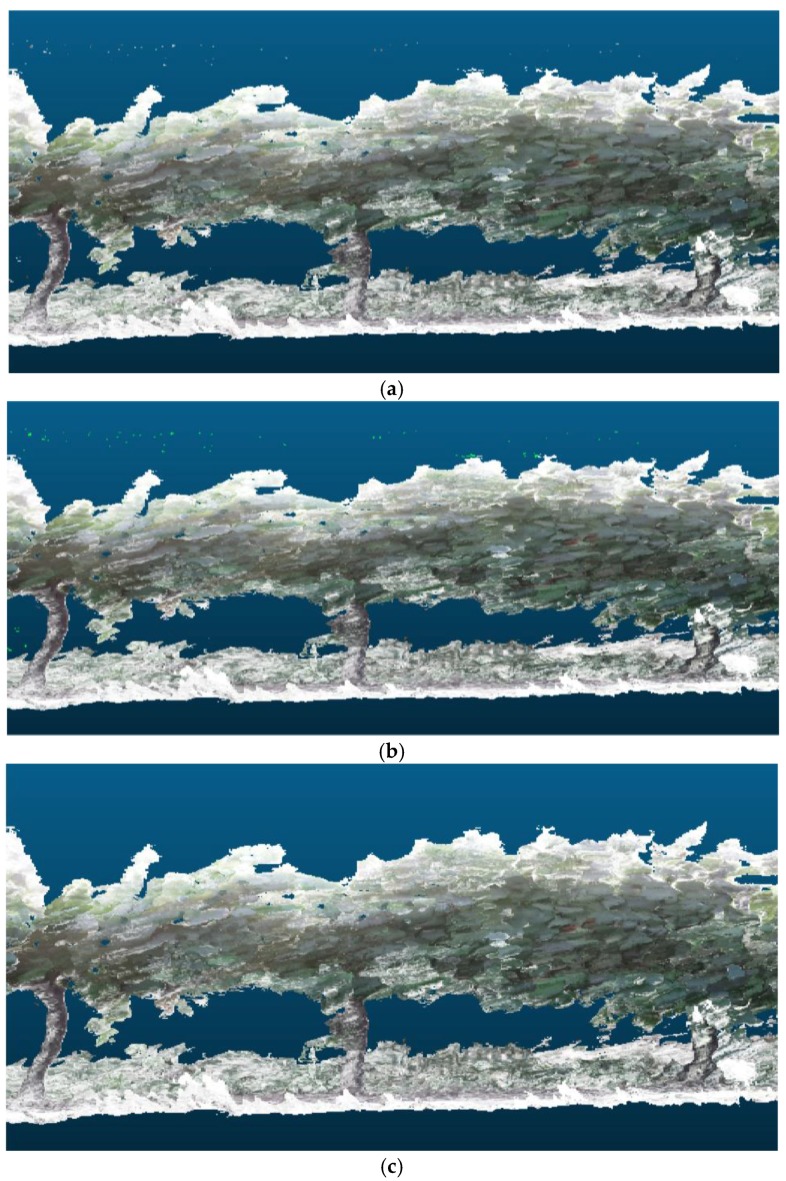
Section of the 3D reconstruction of a vineyard row: before filtering (**a**); and after filtering (**c**). The removed points are marked in fluorescent green in image (**b**).

The first step is to filter the point cloud to eliminate those points that appear isolated. A point is considered an outlier if the average distance to its 64 nearest neighbours is greater than the standard deviation of the distance to the neighbours of all the points. Figure 3 shows a fragment of a reconstruction of a vineyard row before and after the filtering operation.

#### 2.2.2. Estimation of a Model Line

The next step is to find a line that longitudinally models the 3D reconstruction of the previously generated tree row. This model line should be adjusted as much as possible to the path following the tree row and will be used in the next steps to segment the row in sections of the same length. The positions of the point cloud representing the 3D reconstruction of the row are used to model the line.

**Figure 4:**
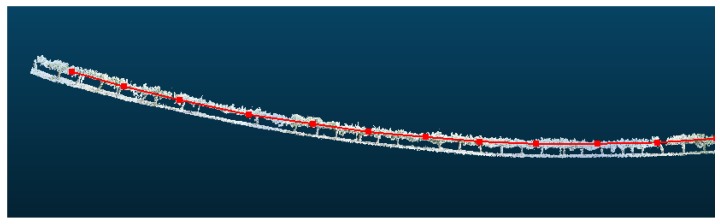
Part of the model line of the 3D reconstruction of Figure 2.

The point cloud is divided into smaller groups of equal numbers of points and the centroids of the points in each group. Since the points in the cloud are stored consecutively according to their order of appearance, the centroids are also consecutive and determine the path followed by the model line. The number of points of the model line is proportional to the number of groups into which the original point cloud is divided, i.e., more groups correspond to more points and a higher accuracy of the fit to the original tree row. The number of groups is estimated from the total length of the tree row and the known geographical position of the starting and ending points of the row. In general, it is sufficient to divide the row into groups of approximately 5 m, as we have experimentally verified that the effect of the drift over 5 m can be considered to have a negligible effect on the performance of the proposed method. Note that the groups of points, although they include the same number of points, do not represent the same row length because the density of points is not necessarily uniform (i.e., the density changes depending on the degree of tree coverage); thus, the points that define the model line are not at the same distance, as shown in Figure 4.

**Figure 5:**
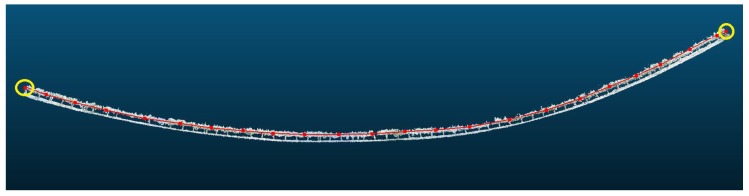
Yellow circles represent the endpoints of the tree row added to the model line of Figure 4.

To obtain the model line that covers the tree row end to end, it is necessary to determine the endpoints of the row to incorporate these endpoints to the points that form the model line. To identify the starting point, the points of the first group are projected onto the extension of the line connecting the first and second centroids. The projected point most distant to the first centroid is chosen as the starting point of the model line since, in a frontal view, all other points of the tree row are to the right of the centroid. The procedure to be followed is similar in the case of the ending point estimation. All points of the last group are projected on the extension of the model line that connects the penultimate and last centroids, choosing as the ending point of the model line the point projected as most distant to the last centroid since all the points of the tree row are to the left of this centroid (Figure 5).

#### 2.2.3. Splitting the Model Line into Sections of the Same Length

Once an accurate model line of the crop is obtained, the line is divided into segments of equal length. The model line length is estimated by adding the Euclidean distances between the points that define the line. This value is compared with the actual tree row length obtained from the geographical position of the starting and ending points of the row. A slight difference is expected due to the drift of the row, which makes the length of the model line greater than the actual length. To distribute the error along the entire tree row, the desired length of the segment is adjusted proportionally to the ratio between the model line length and the actual tree row length. For example, if the actual row length is 50 m, its corresponding model line is 53 m in length, and a scale of 0.5 m is desired; thus, both the actual row and the model line will be divided into 100 sections. Consequently, each actual row segment of 0.5 m will have a corresponding model line segment of 0.53 m to maintain the number of sections, thus distributing the error. Subsequently, when the drift is corrected, the change will be undone, and the segment will again measure the actual 0.5 m.

**Figure 6:**
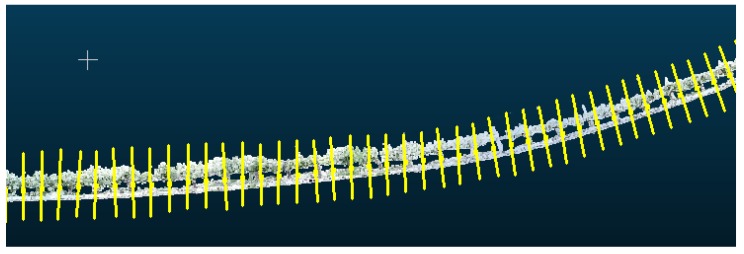
Part of the 3D reconstruction shown in Figure 2 split into sections.

Therefore, once the length of the section is defined, the model line is divided into segments of that length and the 3D reconstruction or point cloud is divided into sections separated by planes perpendicular to the model line at each point of segment separation (Figure 6). In this manner, the point cloud is divided into sections, each containing a fragment of the 3D reconstruction.

#### 2.2.4. Section Correction

**Figure 7:**
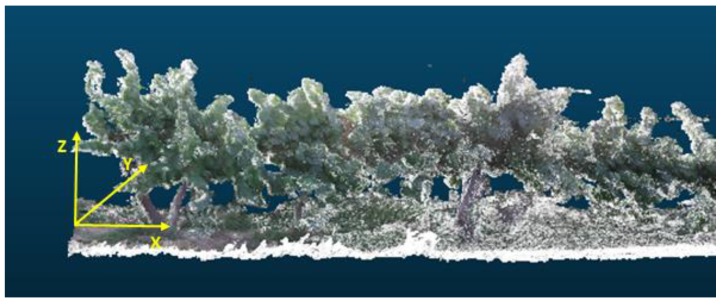
The coordinate system defined in yellow.

First, to facilitate the completion of the developed procedures, the coordinate system must be changed since the origin of the coordinate system for the generated point cloud is placed in the initial position of the Kinect sensor. The applied transformation matrix places the origin in the initial position of the crop, centred in the soil, with the X-axis in the direction of the crop row (sense from the starting point to the ending point of the crop row), the Z-axis in the direction of tree growth, and the Y-axis in the direction of the crop depth (Figure 7).

**Figure 8:**
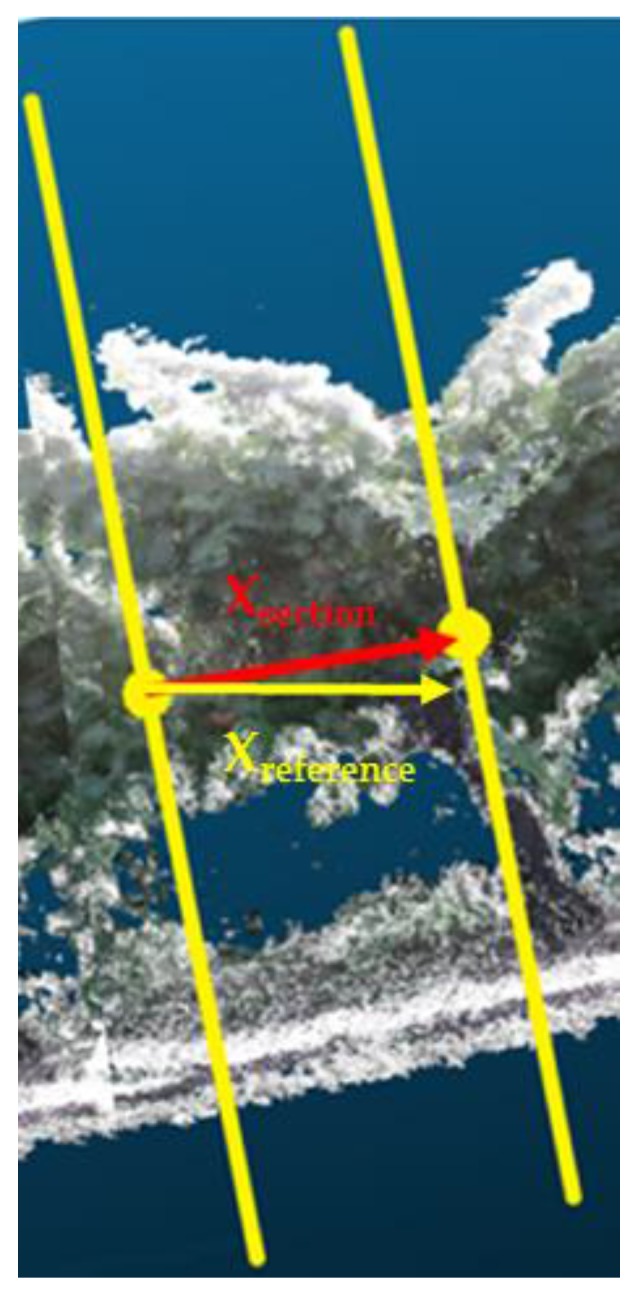
The X-axis of the section (Xsection) is represented in red, and the X-axis of the reference (Xreference) is represented in yellow.

The vector that indicates the direction of the crop row in each section can be calculated using the starting and ending points of the model line segment contained in the section, drawn as a red line in Figure 8. Ideally, the direction of this vector coincides with the direction of the actual tree row (i.e., the reference X-axis represented in Figure 7); however, this condition does not occur due to the drift that appears in the 3D reconstruction. To eliminate the drift, each section must be corrected in such a manner that the section must be rotated to align the vector that indicates the direction of the model line in the reconstructed section (Xsection) with the direction of the X-axis of the actual section (Xreference). To accomplish this correction, first, in each section, the desired reference system is placed at the starting point of the segment in the section by performing a translation from the coordinate origin of the reconstructed row to that point. Next, after normalizing the vector Xsection, the rotation matrix R (see Equation (1)), which rotates the unit vector Xsection to align it with the unit vector Xreference, must be determined, as shown in Figure 8.

Euler’s rotation theorem [24] states that any rotation or set of successive rotations can be expressed as a rotation about a principal axis of rotation or vector (v) that, in this case, can be calculated by the cross product of vectors Xsection and Xreference.

Furthermore, Rodrigues’ rotation formula [25] mathematically states the rotation theorem of Euler, enabling the rotation matrix R calculation, given an axis of rotation v and an angle of rotation θ, as shown in Equation (1):

(1) $$R\;=\;I\;+\;{\left[v\right]}_{\times }\;\cdot \;\sin\;\theta \;+\;{\left[v\right]}_{\times }^{2}\;\cdot \;(1\;-\;\cos\;\theta ),$$

where ${\left[v\right]}_{\times }$ represents the v-axis obtained from the cross product and expressed in terms of a skew-symmetric matrix (Equation (2)):

(2) $${\left[v\right]}_{\times }\;=\;\left(\begin{array}{ccc} \;0 & {-v}_{3} & {\;v}_{2}\\ v_{3} & \;0 & {-v}_{1}\\ {-v}_{2} & {\;v}_{1} & \;0 \end{array}\right),$$

Since Xsection and Xreference are unit vectors, the cosine of the angle between them can be obtained as the dot product of both vectors (Equation (3)):

cosθ = Xsection·Xreference(3)

In a similar manner, the sine of the angle between vectors Xsection and Xreference can be calculated using Equation (4), i.e., as the norm of the axis obtained from the cross product of both vectors:

(4) sinθ=||v||

At this point, all terms necessary to calculate the rotation matrix, R, from the Rodrigues formula (Equation (1)) are available. Applying such a matrix to all the points forming the section, the section is rectified, aligning the direction of the crop in that section with the actual X-axis and thus correcting the drift. With the section correctly placed, the coordinate transformation previously performed is undone so that all section points are referenced with respect to the coordinate origin of the reconstructed row. As explained in Section 2.2.3, once the drift is corrected, the change in the length of the segment is reversed by rescaling the segment such that its initial length is restored.

**Figure 9:**
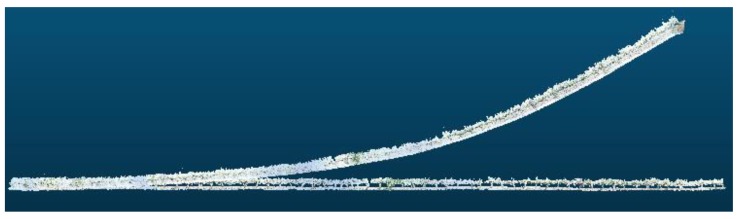
3D reconstruction of a tree row with drift (up) and the result after the drift correction procedure is applied (down).

Once the drift of each section has been corrected, the sections are aligned; thus, the drift produced during 3D reconstruction is corrected. Figure 9 shows a reconstruction of a vineyard row with drift and the result obtained after applying the developed procedure.

## 3. Results and Discussion

**Figure 10:**
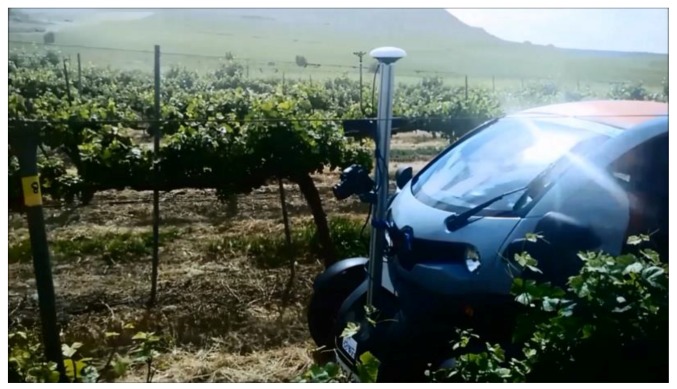
Detail of the sampling in a vineyard using the field platform.

Several tests were conducted in 2016 in vineyards owned by Codorniu S.A. (Raimat, Lleida, Spain). The abovementioned inspection platform moving at 3 km/h was used to collect the information. The sensor Kinect v2 was mounted at approximately 1.4 m high with a 10° pitch angle oriented to the crop rows at a distance of approximately 1 m from the crop row (Figure 10).

From each inspected row, the starting and ending geographical positions of the row supplied by the RTK-GPS receiver from the vehicle were stored.

**Figure 11:**
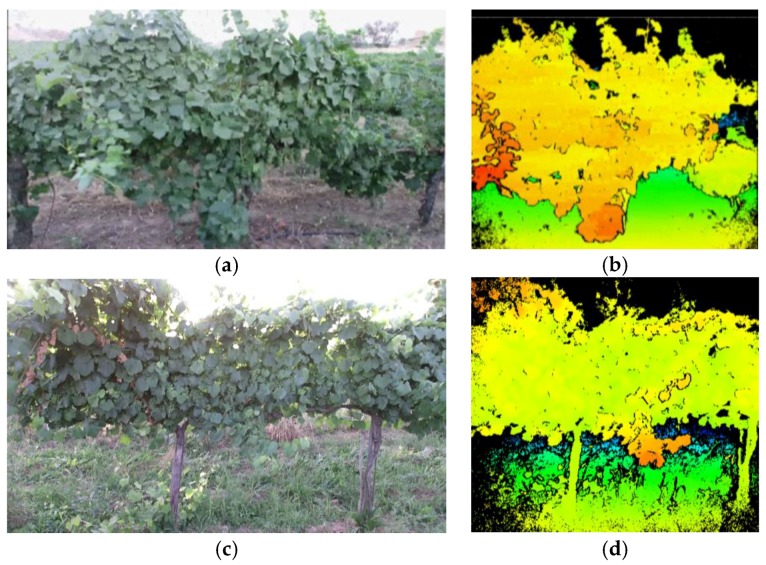
(**a**,**c**) RGB images supplied by the Kinect v2 sensor; and (**b**,**d**) examples of the depth images supplied by the Kinect v2 sensor simultaneous to when images (**a**,**c**) were obtained.

Figure 11 shows examples of the information provided by the Kinect v2 sensor in the vineyard in the tests conducted on May 2016. Figure 11a,c shows the RGB information of two scenes. Figure 11b,d shows a false-colour representation of the depth information, i.e., the distance from the camera to the objects in the scene, where the closest objects appear in red and the furthest objects appear in blue; the intermediate objects are shown in various shades of orange, yellow and green depending on their distance from the sensor. The operating range of the sensor meets the inspection requirements of the vineyard rows since the non-interesting objects are ignored, such as those extremely close and those within distant areas that typically contain other vineyard rows.

**Figure 12:**
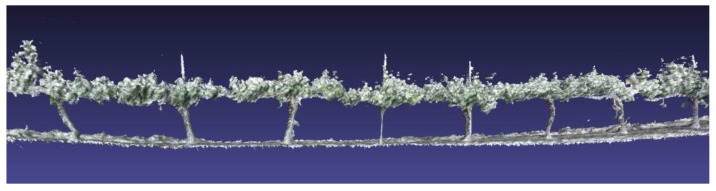
3D reconstruction of a row of vines.

**Figure 13:**
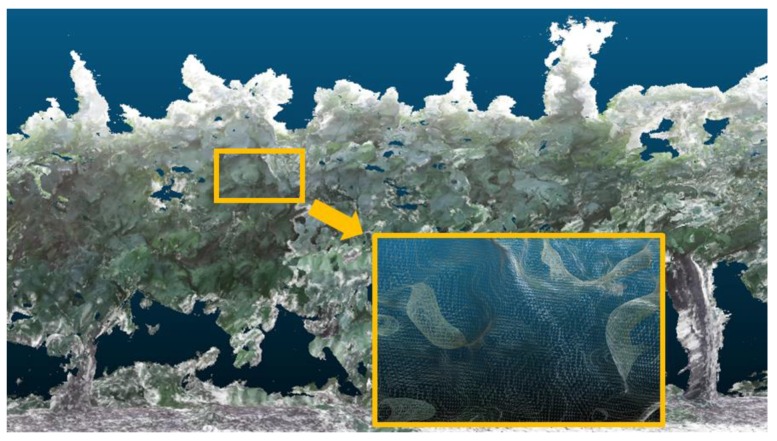
Details of a 3D reconstruction of a vine that shows the triangular mesh obtained from a point cloud.

With the information supplied by the Kinect v2 sensor and stored in the on-board computer of the vehicle, the 3D reconstruction of the sampled rows of vines was performed. For that reconstruction, a desktop computer with an Intel Core i7-6900K@3.2GHz processor, 64 GB of RAM, and an NVIDIA GeForce GTX Titan X graphics card was used. Figure 12 shows an example of one of the 3D reconstructions that includes a 3D mesh structure, i.e., a triangular mesh (Figure 13) obtained from the point cloud using the marching cubes algorithm [26].

**Figure 14:**
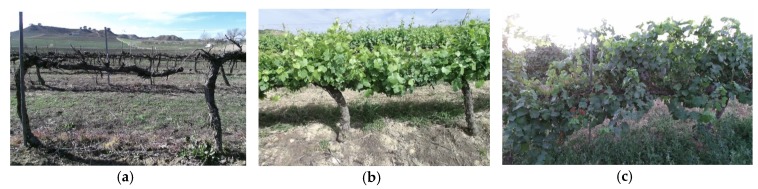
Condition of the vineyards used in the experiments conducted in: (**a**) February 2016; (**b**) May 2016; and (**c**) July 2016.

As discussed earlier, a drift that causes distortion in the row reconstruction typically appears in the 3D reconstructions of long crop rows. To evaluate the performance of the proposed approach to correct the drift, 3D reconstructions of long crop rows sampled at different times of the year were conducted. Figure 14 shows the condition of the vineyard in the tests conducted in February, May and July 2016.

**Figure 15:**
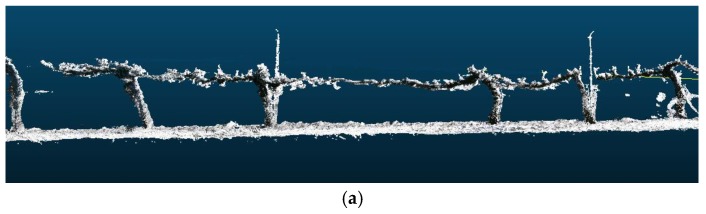

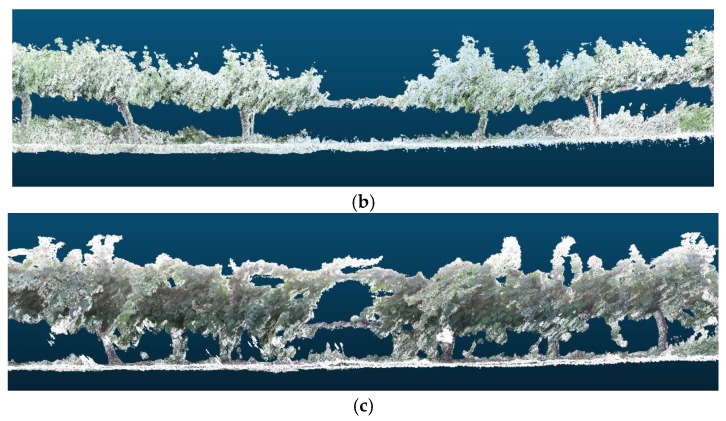
The same view of the 3D reconstruction of a vineyard with the information acquired in: (**a**) February 2016; (**b**) May 2016; and (**c**) July 2016.

Thus, the method was tested with trees at distinct stages of development, in which the structure to be reconstructed was different and the number of points within clouds was smaller or greater depending on the time of year, i.e., without leaves, with leaves, and with both highly developed leaves and vegetation cover. Figure 15 shows the 3D reconstruction of the same part of a vineyard in February, May and July 2016.

**Figure 16:**
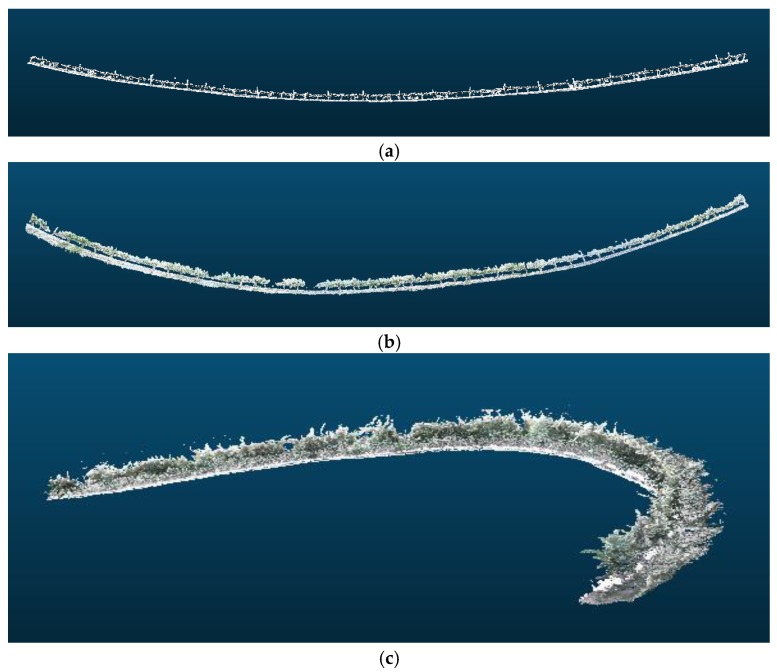
Examples of 3D reconstructions that exhibit drift. Sampling performed in: (**a**) February 2016; (**b**) May 2016; and (**c**) July 2016.

During the several experiments conducted, although 3D reconstructions of long crop rows were properly performed, drift appears in all the tests conducted throughout the year (Figure 16).

**Figure 17:**
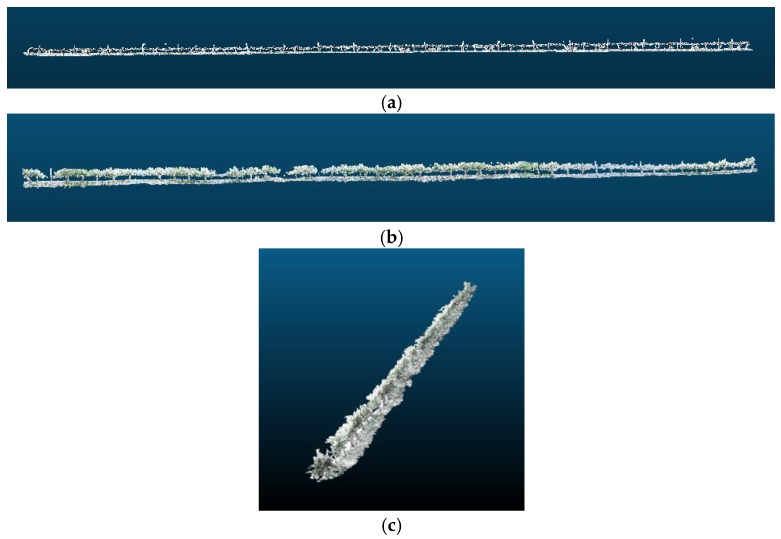
(**a**–**c**) 3D reconstruction of vineyards in Figure 16 after the drift has been removed.

Using the method described before, it is possible to correct the drift. The vineyard row in Figure 16 is divided in sections of 5 m, and the rotation angle of each section is estimated using Rodrigues’ rotation formula to correct the drift. Table 1 shows the calculated rotation angles per section. Thus, using the proposed approach, the drift, shown in Figure 16, was corrected to obtain the images displayed in Figure 17.

**Figure 18:**
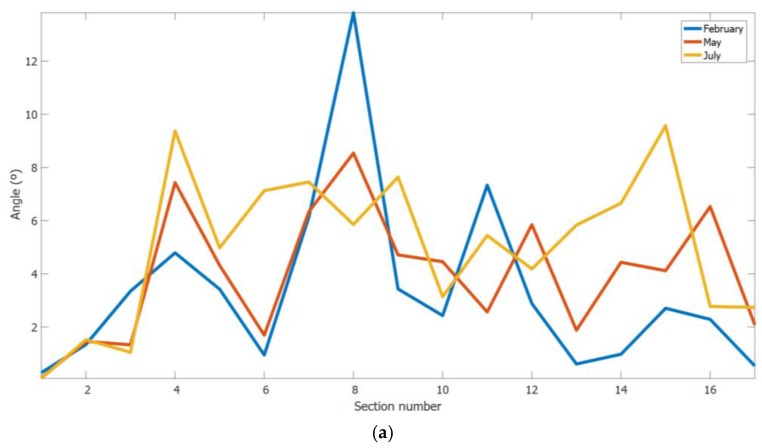

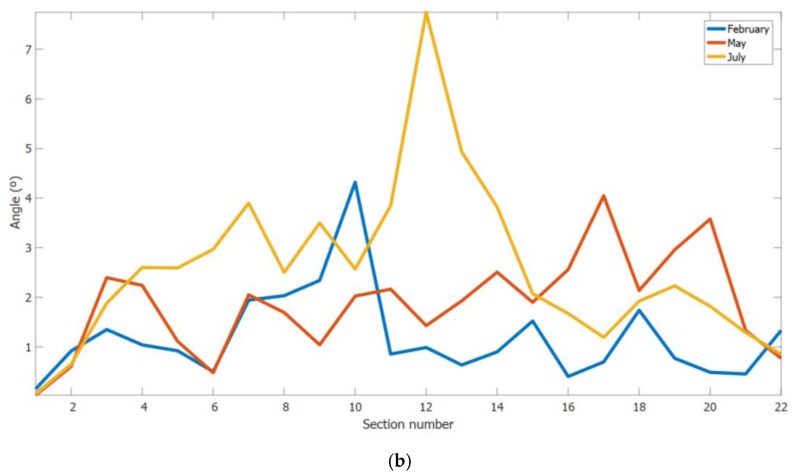
Rotation angles calculated by the proposed approach to correct each section on the vineyard rows of: (**a**) 85 m length; and (**b**) 105 m length. Sampling performed during February, May and July 2016.

Next, the performance of the proposed approach was analysed through a 3D reconstruction of two rows of the vineyard with lengths of 85 m and 105 m. Figure 18 shows the rotation angles estimated to correct each section on the rows. Table 1 and Table 2 show the mean, standard deviation and maximum of the calculated rotation angles of all the sections for the two considered rows and the number of points of the cloud and the length of the model line that defines each row. From the results, it can be concluded that a smaller number of leaves in the vineyards is correlated with a smaller deformation in the 3D reconstructions. This behaviour arises because the ICP algorithm, which estimates the position and orientation of the Kinect sensor, is not as effective when the same structure is repeated, i.e., the appearance of similar leaves results in analogous distances. It seems that, when the length of the row to be reconstructed is greater, the deformation that appears in the 3D reconstruction is greater because of the error accumulated along the row. This fact must be confirmed through a specific and deeper analysis.

Additionally, the processing time to correct the drift depends on the number of the points of the cloud. To reduce this time, one option is to perform a uniform downsampling of the point cloud. For example, when the downsampling percentage equals 75%, the original point cloud is sampled uniformly, preserving 25% of the points. It may appear that, if the resolution to generate the 3D reconstruction were reduced, the effect would be the same. However, in this case, the ICP algorithm performs worse, and the drift of the 3D reconstruction increases.

To study the influence of downsampling, differences among the calculated rotation angles applied to each section in the clouds with a distinct downsampling percentage were calculated. Taking the correct values of the angles as those calculated with 100% of the points, the errors among this value and those obtained values with the downsampled point clouds were obtained. Table 3 shows the mean error of all sections, the maximum error in these sections, and the processing time to correct the drift in the computer described above for different downsampling percentages of the point cloud analyses.

Using 50% of the points, the maximum error is less than 0.2°. When downsampling is performed at 75%, the maximum error is tripled (to 0.61°), although this error is still within an acceptable range of error. Performing a downsampling at 90%, the error further increases until it becomes inappropriate for a proper drift correction, since it is greater than 1°.

When the number of points of the cloud is greater, the effect of downsampling on the processing time is greater. The relationship among the number of points and the processing time is not linear; for example, comparing the processing time downsampling at 75% with respect to the original point cloud, in the cloud with fewer points (February, 85 m), the processing time is reduced by a factor of 2.34, and, in the cloud with the highest number of points (July, 105 m), the processing time is reduced by a factor of 4.17.

For certain applications, it may not be necessary to use such a high number of points (such as measuring the height of the tree), and fewer points would suffice, thus enabling the drift correction process to be accelerated without producing high errors. If the use of all points is required but the process must be accelerated, one solution is to perform the drift correction algorithm on the downsampled point cloud at 75%. In other words, the planes, which delimit each section, and the angle applied to the section to correct it are established via a calculation in the reduced point cloud for later use in the complete point cloud. This strategy could reduce the processing time by a significant amount without considerably increasing the error.

## 4. Conclusions

This paper describes a low-cost crop monitoring system that can automatically create accurate 3D models of woody crop rows. The system integrates a medium-size platform equipped with various on-board sensors to scan annual crops (maize, cereal, etc.) and multi-annual crops (orchards, vineyards, etc.). It also incorporates software specifically developed to accurately generate 2D and 3D maps of the sampled crops. Particularly, the paper is focused on the automatic 3D reconstruction of woody crops based on the information obtained from an on-board RGB-D sensor.

Based on the results of the samplings conducted, it can be concluded that the implemented algorithm provides good results for the automatic 3D reconstruction of large areas under uncontrolled lighting conditions at different times of the year within commercial vineyard fields. Furthermore, the operating range of the sensor meets the inspection requirements of the vineyard rows since the non-interesting objects are ignored, including objects extremely close to the sensor and those at distant locations that usually correspond to other vineyard rows. The drift that usually appears in the 3D reconstruction of long rows, those greater than 25 m, can be properly handled with a method that uses information about the scene, such as the geographical position of the starting and ending points of the row and the fact that the woody crops are typically planted in straight rows. Additionally, a smaller number of leaves in the vineyards is correlated with a smaller deformation in the 3D reconstructions. The processing time to remove the drift depends on the number of points in the 3D reconstruction and can be reduced by performing a uniform downsampling of the point cloud. Moreover, it has been verified that the greater the number of points in the cloud, the greater the effect of downsampling on the processing time. Finally, it can be concluded that in some cases, it might not be necessary to use such a large number of points in the removal drift process, accelerating the process without producing high errors.

Considering future work, the developed drift removal method should be able to properly handle field crops that are planted in curved rows. A potential strategy might be to suitably incorporate the information of all the GNSS locations taken during the sampling. Additionally, the integration of an inertial measurement unit (IMU) fixed to the Kinect sensor for registering variations in the yaw, pitch and roll angles of the sensor could be a good strategy to obtain complementary and useful information for improving the 3D reconstruction accuracy.

## Figures and Tables

**Table 1:** Statistics of the performance of drift correction method for the 85 m length row.

Sampling	Number of Points	Mean Angle (°)	Std. Dev. Angle (°)	Maximum Angle (°)	3D Reconstruction Time (s)	Model Line Length (m)
February	4,289,633	3.56	3.33	13.82	42.46	86.30
May	6,441,484	3.99	2.42	8.54	64.03	89.69
July	13,624,626	5.02	2.83	9.57	83.76	93.81

**Table 2:** Statistics of the performance of drift correction method for the 105 m length row.

Sampling	Number of Points	Mean Angle (°)	Std. Dev. Angle (°)	Maximum Angle (°)	3D Reconstruction Time (s)	Length of Model Line (m)
February	5,575,934	2.39	1.80	8.63	60.52	107.21
May	7,941,965	3.72	1.96	8.09	72.85	112.00
July	16,000,906	5.39	3.31	15.49	111.09	115.93

**Table 3:** Different measures for different downsampling percentages of the point clouds studied.

Crop Row	Sampling	Measures	Downsampling Percentage					
			0%	50%	75%	90%	99%	99.9%
		Mean error (°)	-	0.06	0.18	0.27	0.97	1.30
85 m	February	Max. error (°)	-	0.19	0.61	1.30	3.69	3.85
		Time (s)	119.25	58.30	50.59	12.65	2.09	0.99
		Mean error (°)	-	0.05	0.09	0.11	0.23	0.75
85 m	May	Max. error (°)	-	0.17	0.29	0.30	0.68	2.54
		Time (s)	213.60	89.78	65.06	18.44	2.59	1.11
		Mean error (°)	-	0.01	0.03	0.10	0.39	0.63
85 m	July	Max. error (°)	-	0.04	0.14	0.41	1.72	2.07
		Time (s)	389.49	183.65	93.38	36.04	4.62	1.67
		Mean error (°)	-	0.03	0.05	0.09	0.34	0.42
105 m	February	Max. error (°)	-	0.10	0.22	0.26	1.61	1.61
		Time (s)	155.01	75.77	65.76	16.43	2.73	1.21
		Mean error (°)	-	0.05	0.05	0.16	0.40	0.85
105 m	May	Max. error (°)	-	0.18	0.10	0.60	1.49	3.37
		Time (s)	263.35	110.71	85.92	22.64	3.18	1.45
		Mean error (°)	-	0.04	0.11	0.26	0.33	2.34
105 m	July	Max. error (°)	-	0.15	0.38	0.90	0.96	13.55
		Time (s)	457.52	214.68	109.66	42.23	5.32	1.97
